# Attitudes and Barriers to the Use of Telemedicine in the Ultra-Orthodox Society in Israel: A Cross-Sectional Study

**DOI:** 10.3390/ijerph23030381

**Published:** 2026-03-17

**Authors:** Shira Ramot, Galia Barkai, Galit Hirsh-Yechezkel, Angela Chetrit

**Affiliations:** 1 Gertner Institute for Epidemiology and Health Policy Research, Sheba Medical Center, Ramat Gan 5266202, Israel; galith@gertner.health.gov.il (G.H.-Y.); angelac@gertner.health.gov.il (A.C.); 2 Sheba BEYOND, Sheba Medical Center, Ramat Gan 5266202, Israel; galia.barkai@sheba.health.gov.il; 3 Gray Faculty of Medical & Health Sciences, Tel-Aviv University, Tel-Aviv 6997801, Israel

**Keywords:** telemedicine, ultra-orthodox society, health disparities

## Abstract

**Highlights:**

**Public health relevance—How does this work relate to a public health issue?**

- This study identified factors and barriers influencing the use of telemedicine among the Ultra-Orthodox population in Israel, a minority group with unique cultural constraints.
- Telemedicine can improve healthcare access but at the same time, it may also widen public health disparities among minority populations, especially those who face cultural or religious barriers and limited access to technology like the Ultra-Orthodox population.

**Public health significance—Why is this work of significance to public health?**

- Despite cultural and technological challenges, the study’s findings reveal that telemedicine is being used within the Ultra- Orthodox community, with phone consultation, being the most common use of telemedicine services -indicating its potential as an accessible modality for populations facing cultural or technological barriers.
- The main barrier to the use of telemedicine among the research participants is a religious-ideological reason, and about a third of the respondents stated that they do not have a smart phone or internet- posing a specific public health challenge.

**Public health implications—What are the key implications or messages for practitioners, policy makers and/or researchers in public health?**

- The study findings may assist policy makers and healthcare systems in expanding the provision of telemedicine services within the Ultra-Orthodox community, for example, by further developing and scaling telephone-based telemedicine services for this population in Israel.
- The finding underscores the need for careful consideration when implementing telemedicine services within the Ultra-Orthodox community, through the development of culturally tailored solutions and strategies that align with its unique characteristics and may enhance motivation to use telemedicine.

**Abstract:**

The use of telemedicine by the Ultra-Orthodox (UO) population in Israel presents challenges due to unique cultural characteristics, including limited internet use for religious ideological reasons and lower levels of digital literacy. This cross-sectional survey examines the rate of telemedicine use in the UO society in Israel according to religious groups, factors, attitudes and barriers associated with telemedicine use. The study included 1460 adult UO participants using quota by gender, and religiosity groups. The participants underwent a phone interview assessing telemedicine use (defined as at least one monthly phone/video call/e-mail correspondence with a medical professional, during the last year), attitudes, and perceived barriers. In total, 39% of participants used telemedicine and 42% performed one or more administrative actions. Phone consultations were the most common mode of communication with healthcare providers. The main barrier to using telemedicine was religious-ideological. Multiple logistic regression revealed that female sex, participants aged 30–44, married status, above-average income, frequent family physician visits, and internet use significantly associated with telemedicine use. Compared to <30, adults aged 60+ years use less telemedicine (OR 0.52, 95% CI 0.32–0.86). These findings indicate telemedicine use within the UO population, though substantial cultural barriers remain, and may assist policymakers in expanding its implementation.

## 1. Introduction

Telemedicine represents a significant advancement in healthcare, delivering notable improvements in preventive services and the management of chronic conditions. Moreover, the COVID-19 pandemic has accelerated and catalyzed the implementation and use of telemedicine which enabled continuity of care along with maintaining social distancing measures and preventing virus exposure for the patient and staff. WHO defined telemedicine as the delivery of healthcare services, where distance is a critical factor, by all healthcare professionals using information and communication technologies for the exchange of valid information for diagnosis, treatment and prevention of disease and injuries, research and evaluation, and the continuing education of healthcare workers, with the aim of advancing the health of individuals and communities [1]. It may be used through different modalities, including video, audio calls, text messages and smartphone applications encompassing a wide range of services including online patient consultations, telehealth nursing, and remote physical and cognitive rehabilitation. Telemedicine offers numerous advantages, including time and cost saving for patients and increasing accessibility for the elderly, disabled patients, and geographically distant patients [2]. Furthermore, telemedicine has been shown to reduce waiting times for patients compared to traditional in-person appointments [3,4]. Despite these benefits, several obstacles persist like patient privacy loss, technical literacy, patient safety, and ethical concerns [5]. Specifically, technology barriers and lack of computer literacy prevailed as a major issue in successfully implementing telemedicine [6]. Moreover, telemedicine may lead to an increase in social and health inequality, especially since ethnic minorities, those with low education, weak populations and those with limited access to technology and low digital literacy, have less access to telemedicine [7,8,9,10].

When implementing telemedicine services, it is essential to ensure that the delivery of care is responsive to the cultural and ethnic needs of diverse patient populations. Telemedicine needs to be trusted and usable, equitable and accessible across all populations [11]. Certain populations have unique challenges in accessing telemedicine like individuals with limited digital literacy or those who lack access to digital devices [12]. One of the populations in Israel that requires special consideration in the context of telemedicine is the Ultra-Orthodox population (Haredi). This minority is a closed religious community, which seeks to be isolated from many influences of modernity [13]. Rabbis are regarded as highly authoritative figures and have control and influence over all aspects of life, such as family life, voting and education, as well as technology use [14].

The Ultra-Orthodox society is characterized by a low average age at marriage and a high fertility rate, contributing to rapid growth over the last two decades [15]. The Israeli Central Bureau of Statistics anticipates they will triple over the next three decades to up to 30% of the total population of Israel by 2050 [16]. This society is heterogeneous comprising three main subgroups: Hasidic, Lithuanian and Sephardic [17] which differ in several aspects such as community membership, rabbinical leadership, worldview, educational system, and attitude towards types of livelihoods. Generally, the Lithuanian and the Sephardic group demonstrate greater openness toward modern lifestyle integration compared to the Hasidic group [18].

One of the main conflicts of the Ultra-Orthodox society is to what extent to open up to the surrounding environment (the broader secular and modern world) and its influences. In recent years, this population is undergoing accelerated changes resulting from its unavoidable interaction and integration with wider society [19]. While health is a paramount religious obligation [20], hospitalization and encounters with secular society raise concern about preserving their religious lifestyle and communal identity [21]. Traditionally, due to a complex relationship with modernity, technologies in general and the internet in particular, have been perceived as threats to the community’s conservatism. There are ambivalent attitudes towards it- in some parts of the Ultra-Orthodox society using the internet is forbidden, while in others the use is permitted in a limited and adapted manner. Consequently, the Ultra-Orthodox society is in a big gap from the general population in Israel, both in the level of consumption of online services and in digital literacy [22].

However, in recent years the use of the internet has increased, and members of the Ultra-Orthodox society are integrating internet use into their daily lives. This trend is driven by a growing number of individuals entering the workforce and higher education, alongside shifting patterns in leisure and consumption characteristics within Ultra-Orthodox culture [15]. In 2018–2019, a significant majority of the adult Ultra-Orthodox population reported using the internet for the first time [15] (54% compared to 90% among the non-Orthodox Jewish public). The COVID-19 pandemic accelerated these processes of increase in use. In an Israeli survey conducted in the midst of the COVID-19 pandemic (4/2020) among the Ultra-Orthodox population, 78% reported that they browse the internet more than before [23]. A 2020 survey of 678 respondents revealed that 66.2% of the Ultra-Orthodox population use the internet, while 65.5% reported having a computer at home. Furthermore, 44% of the participants, representing 80,000 households, had a home internet connection. Differences were found between groups in the Ultra-Orthodox sector regarding computer ownership and internet connectivity [24]. The mode of access also differs from the general population: Ultra-Orthodox individuals tend to connect mainly via their home computers (42%), rather than through mobile phones (30%).

Although there is an increase in internet use among members of the community, its use is still limited, and many households refrain from using it [25]. Accordingly, the implementation of telemedicine in the Ultra-Orthodox society poses many challenges, including their hesitation to participate in this new mode of care delivery that may be viewed as conflicting with their religious principles [12]. Thus, integrating telemedicine into the Ultra-Orthodox society requires adjustments including identifying and mapping the barriers and needs of the society in order to make the service optimally accessible to this population. To the best of our knowledge, the information on the use of telemedicine in the Ultra-Orthodox society in Israel is limited. Consequently, this study was conducted to evaluate the rate of telemedicine use in the Ultra-Orthodox society according to religious groups (Hasidic, Lithuanian and Sephardic), identify the factors associated with its use, and map the primary barriers to implementation within this unique population.

## 2. Materials and Methods

### 2.1. Setting and Participants

This cross-sectional survey included a sample of 1460 participants from the Ultra-Orthodox society constituting a representative sample of the adult Ultra-Orthodox population in Israel.

### 2.2. Study Tool

The study questionnaire included participants’ sociodemographic characteristics, health status and regular use of health services, as well as internet usage and accessibility to digital devices. In addition, respondents were asked about their use of telemedicine in the previous year, including questions regarding attitudes and barriers to the use and willingness to use telemedicine.

### 2.3. Data Collection

Data collection was carried out through a phone interview during 2023 by the Askaria survey institute, which specializes in the Ultra-Orthodox population. The sample was drawn from the company’s databases, which cover over 85% of the Ultra-Orthodox sector using quota-sampling techniques to ensure that the sample represented the Ultra-Orthodox society with respect to gender and religiosity groups.

### 2.4. Statistical Analysis

Telemedicine was defined as having a phone/video call or e-mail correspondence with a medical professional. Use was categorized as: frequent (at least one monthly encounter during the last year prior to the interview) and seldom/no use (less than monthly or no interactions).

Student’s *T*-test and the nonparametric Mann–Whitney test were performed in order to evaluate differences in socio-demographics characteristics and additional study variables between interviewed and non-interviewed; and between telemedicine frequent users and seldom/no user groups for continuous variables. Categorical variables were compared using the chi-square test or Fisher’s exact test for small cells.

The peripherality levels are based on indices developed by the Israel Central Bureau of Statistics based on the distance from opportunities (such as markets, employment centers, and health services), from activities (such as work, education, shopping), or from assets available in all areas, including within the area itself. According to its index values, each locality in Israel is classified as one of 10 clusters of peripherality (where cluster 1 includes the most peripheral localities and cluster 10 includes the least peripheral ones). Central localities are defined as levels 7–10. The house density index was calculated by dividing the number of residents by the number of rooms (where <1 means low level of density and >1 means high level of house density). Three types of mobile phones were defined: “Kosher” smartphone—internet- and apps-restricted smartphone, regular smartphone and a simple mobile phone (not smartphone).

Multiple logistic regression models were used to assess factors associated with telemedicine use, where the reference category was set for no telemedicine visits. Socio-demographic variables, use of healthcare services, and accessibility to the technology were entered as separate blocks in the multivariable models. All variables examined in univariate analysis that were found to be significantly associated with telemedicine use were entered into the models (the cutoff for variable inclusion was *p* = 0.10) and backward elimination was employed to remove variables that did not associate with the outcome. Age, sex and religious groups (Hasidic, Lithuanian and Sephardic) were retained in the models, and odds ratios with 95% confidence intervals (95% CI) were presented. The variables entered in the model were family status, income levels compared to the national average income of the Ultra-Orthodox population (much below the average, below, similar and above), family doctor visits in the last year, personal mobile phone and internet use.

Multi-collinearity between variables regarding accessibility to technology (computer-desk, laptop, internet subscription, internet use and personal mobile phone) was tested using Cramer’s V statistic and covariates that showed high correlation (≥0.6) were not both retained in the model.

Possible interactions were examined between religious groups and variables of accessibility to technology by incorporating a cross-term product of religious groups with the selected variables in the multivariable regression model.

Analyses were conducted using SPSS software (version 27) and statistical significance was defined as a two-sided *p*-value of <0.05.

## 3. Results

### 3.1. Sociodemographic Characteristics of the Study Population

A total of 2022 participants were contacted, of whom 1460 completed the interview, yielding a response rate of 72.2%. Gender, age, marital status and religiosity group were compared between interviewed and not interviewed and revealed no statistical differences in mean age (35.8 vs. 34.8) and marital status (80% vs. 80.2%) respectively. Regarding religiosity, the interview rates were comparable across the 3 groups: 27% among Hasidic, 24.8% among Lithuanian and 28.8% among Sephardic participants, (*p* = 0.3). Higher proportion of males were found among the non-interviewed compared to the interviewed participants (56% vs. 49%, respectively, *p* = 0.01).

**Figure 1:**
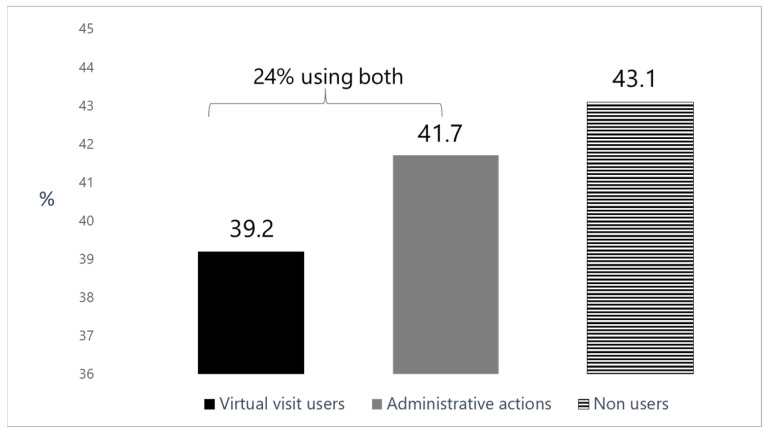
Telemedicine use: virtual visits, administrative actions, and non-users.

Out of the respondents, 743 (51%) were women, 82% were married and 15% single. The participants’ sociodemographic characteristics are summarized in Table 1. As expected from the sampling method and similar to their prevalence in the Ultra-Orthodox population, each religious group accounted for about one third of the cohort. Participants’ mean age was 35.8 ± 14.0 years (range: 18–90) with 75% of the respondents aged less than 45 years. More than 40% of the participants completed yeshiva school without an academic degree and 30.8% have an academic education. About 30% of the participants reported that their income is much below average, 24% reported below average income and 9% refused to answer. In total, 57% of the respondents used telemedicine, 39.2% of them reported using telemedicine (phone call/video call/e-mail) and 41.7% performed online administrative actions (24% used both) at least once a month during the last year. (Figure 1). In addition, 4.2% of the respondents reported using telemedicine at least once a week and 14.7% performed one of the actions of telemedicine several times a month.

**Figure 2:**
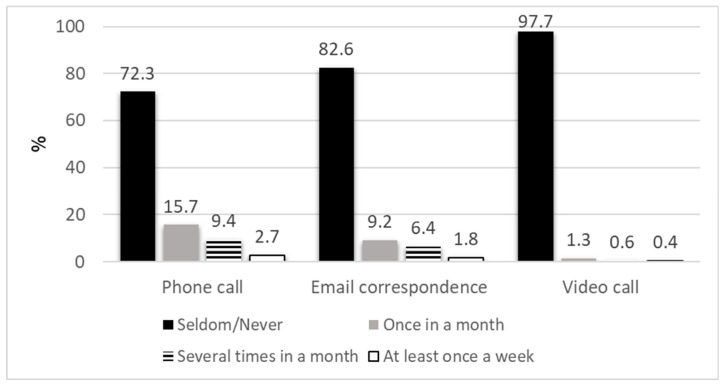
Frequency of telemedicine consultation with medical professionals in the last year by type of service.

As can be seen from Figure 2, 27.8% of the respondents reported consulting with a healthcare provider over the phone, 17.4% by email and only 2.3% by video chat at least once a month during the last year. Other online services such as appointment scheduling (32.6%), accessing personal medical information (31.1%), sending administrative requests by email (21.8%) and ordering medications (9.6%) were performed by 42% of the participants. 

### 3.2. Characteristics of Telemedicine Users

Compared to frequent telemedicine users, seldom/non-users were significantly older (28% vs. 21.6% aged 45 and above, *p* = 0.007), with a greater proportion of single individuals (20% compared to 7.5%, *p* < 0.001) and lower mean number of children (3.9 vs. 4.5, *p* < 0.001) (Table 1). Although no statistically significant difference was observed regarding religiosity group between frequent users, seldom/non-users, a larger proportion of users was reported by the Lithuanian respondents (36% compared to 30% among Hasidic and Sephardic each, respectively, *p* = 0.2). Education and income significantly associate with telemedicine use, expressed by a larger proportion of frequent users with academic education (34.3% compared to 28.5% among non-users) and with above average income (22.6% compared to 14.6% among non-users).

Significant differences were found between frequent users and seldom/non-users regarding the use of healthcare services (Table 2). Among those who reported visiting a family doctor or expert doctor 3–5 times or more than 5 times in the past year, there were more frequent telemedicine users compared to seldom/non-users. Only 9% of the participants reported having a chronic disease with no differences between the study groups.

Regarding the use of technology, significant differences were found between the two groups (Table 3). Those who have a laptop or the internet use more telemedicine compared to seldom/non-users (58.7% vs. 51.1%, *p* = 0.004; and 77.8% vs. 62.3%, *p* < 0.001, respectively). In addition, 22.5% of frequent users have smartphones compared to 17.9% among seldom/non-users, *p* = 0.08. A high frequency of daily mobile phone use was also found among frequent users compared to seldom/non-users; 17.9% of frequent users reported using mobile phone for more than 5 h per day, compared to 13.1% of the seldom/non-users, *p* = 0.01. No indication for multi-collinearity between variables regarding accessibility to technology (computer-desk, laptop, internet subscription, internet use and personal mobile phone) was found.

### 3.3. Comparison of Technology Use Between Ultra-Orthodox Groups

Differences were found between the various religious groups in the type and frequency of technology use (Table A1 in Appendix A). A higher percentage of respondents from the Lithuanian group owned a desktop computer compared to the Hasidic and Sephardic religious groups (31.7%, 20.6% and 22.9%, respectively), owned a laptop (67.7% compared to 38.4% and 55.2% respectively), internet subscription (56.7% compared to 39% and 38% respectively), or had a kosher smartphone (11.9% compared to 8.5% and 10% respectively).

Table 4 presents the factors associated with telemedicine using a multivariable logistic regression model. Women have 70% higher odds of using telemedicine compared to men (95% CI 1.35–2.15). Age shows a clear trend where younger adults (30–44) are more likely to use telemedicine compared to the 18–29 reference group (OR 1.59, 95% CI 1.20–2.11), while older adults (60+) are significantly less likely (OR 0.52, 95% CI 0.32–0.86). Marital status is strongly associated with telemedicine use, with married individuals having more than double the odds compared to unmarried participants and among religious groups, Lithuanian Ultra-Orthodox individuals show marginally higher odds of telemedicine use compared to the Hasidic reference group. Income shows a gradient effect—those with above average income have 71% higher odds of telemedicine use compared to those with much below average income. Healthcare utilization is a strong predictor: people with more than 5 family doctor visits have over three times the odds of using telemedicine (OR 3.23, 95% CI 2.13–4.90) compared to those with no visits. In addition, internet use is significantly associated with telemedicine adoption (OR 2.01), though interestingly, having an internet subscription appears slightly negative (although not statistically significant).

### 3.4. Barriers of Using Telemedicine

**Figure 3:**
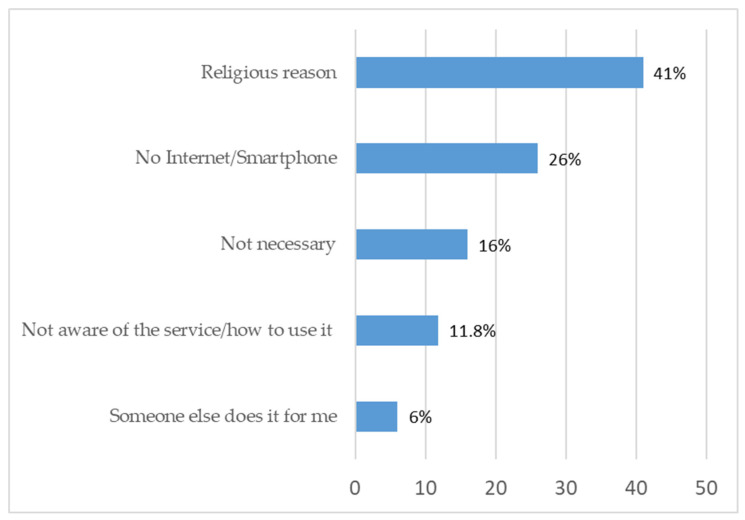
Distribution of barriers of using telemedicine.

The main barrier to the use of telemedicine among participants was a religious-ideological reason (41%). In addition, 26% of the respondents stated that they do not have a smartphone or the internet, 16% stated that they do not need this service and 11.8% did not know about the service or did not know how to use it. When asked what factors could facilitate usage, 31% of the respondents answered -“a kosher device approved by rabbis” (this refers to a device only for telemedicine use that cannot be used online for other purposes). Additionally, 11% specify a device where you can see the doctor on video while 9% stated that if there was an intermediary organization that would provide them with the device and training such as an Ultra-Orthodox Charitable organization, it would allow the use of the service. Another proposed solution was a designated internet room in public spaces (6%) (Figure 3). 

## 4. Discussion

The current study showed a 39% performance of telemedicine visits with a healthcare professional in the Ultra-Orthodox society and 42% use of telemedicine for administrative functions at least once a month during the year preceding the survey. The main barrier to the use of telemedicine among the research participants is religious-ideological. The strongest predictors appear to be female gender, being married, frequent doctor visits, and internet use-suggesting that both digital technological access and specific demographic/health needs drive telemedicine adoption.

Studies have highlighted differences and lower utilization of telemedicine among cultural and ethnic minorities, the elderly, socioeconomically disadvantaged groups, and individuals with limited digital literacy or connectivity [26,27,28].

When comparing the telemedicine use rate found in this study with others in the field, it is essential to consider variations in the definition of a telemedicine user and in the characteristics of the population being studied. Some studies define a telemedicine user broadly as anyone who had at least one of the following with their physicians during the study period: phone/video visit/use of an asynchronous telemedicine service [29]. In contrast, others apply a narrower definition, including only audio-video interaction [30]. Based on several studies, there is an indication that this society uses telemedicine less than the general Jewish population. A study examining telemedicine use in Israel during the COVID-19 pandemic period reported an 85% users’ rate among the Jewish population (users defined as ≥1 phone/video visits or asynchronous encounters during the study period.) The same study reported a lower use of telemedicine in the Israeli minority Arab population, compared to the Jewish population (52% and 85%, respectively) [29].

A 2024 survey with a representative sample of 2558 Israeli adults found no significant difference between Ultra-Orthodox and non-Orthodox Jews in telemedicine consultations (defined as phone call/video call or chat (synchronous)). In contrast, accessing personal medical information and contacting through websites were less used by the Ultra-Orthodox Jewish compared to non-Orthodox Jewish public (54–74% for accessing medical information and 47–63.7% for website-based contact, respectively) [31]. While 39.4% of seniors (≥65 years) utilized telemedicine during working hours [32], only 26% of older Ultra-Orthodox participants of the present study reported using telemedicine consultations. Access to technology and internet connectivity are fundamental requirements for telemedicine [33], and particularly relevant to minority groups such as the Ultra-Orthodox society. Seeking to preserve its identity and resist modern influences, this community often restricts exposure to secular media [32,34]. Their hesitation or unwillingness to use online technology could be a barrier to telemedicine use. As noted, a substantial gap exists between the Ultra-Orthodox and the general Israeli population in both digital literacy and online service consumption despite a recent trend of increased internet use.

The findings of the current study confirm this trend with most participants reporting internet use (67.9%), consistent with other representative studies of the Ultra-Orthodox population in Israel [24]. In our study, most of the participants do not have a smartphone but a simple internet-restricted phone, less than half have an internet subscription, and 54% have a laptop. These results are in line with a survey that examined internet usage patterns among the Ultra-Orthodox in Israel, reporting that 40.4% used the internet via a computer, while only 14.7% used the internet on a mobile phone [35]. In regard to expanding the use of telemedicine services to this population, the pattern of using the internet through a computer more than a smartphone should be kept in mind.

As mentioned, the Ultra-Orthodox society is not homogeneous, and our study found differences in technology use among various subgroups. Respondents from the Lithuanian group reported higher rates of desktop, laptop, and smartphone ownership as well as internet subscriptions, compared to Hasidic and Sephardic participants. Several studies have also found that the Lithuanian group use the internet more than the other Ultra-Orthodox groups [24]. Phone consultation was the most utilized telemedicine service in the current study. This is expected as phone calls are more accessible and simpler to use, particularly given the Ultra-Orthodox society’s ideological barrier toward using online technology. This is also in line with previous studies in the world [36], as well as in Israel [37].

Individuals who use telemedicine exclusively via audio-only tend to be older, adults from ethnic minorities, with lower incomes and education levels, or those living in rural areas, or live in areas with less broadband internet access [38]. Similar patterns emerged during the COVID-19 pandemic, where lower use of video vs. phone consultations was found among older, non-white, low income and rural patients [39]. Consistent with these findings, where 28% of our participants utilized phone consultations, in an Israeli study among the Israeli Arab population, it was found that telemedicine use consisted primarily of telephone appointments [37]. Expanding the service of consulting with a healthcare provider by phone call in the Ultra-Orthodox society can be a solution for the use of telemedicine without the challenge of using the internet and the lack of accessibility.

The results to date on the association between gender and telemedicine use are inconsistent [40,41]. Research studies conducted in Israel reported higher usage of telemedicine among women, in both Jewish and Arab populations [29,37]. Women frequently manage family healthcare and are more engaged than men in contacting providers on behalf of the other family members. Ultra-Orthodox women, who are caring for large families (6.4 children on average) [42,43], work outside the home in order to support their families and their studying husbands and who are exposed to the internet through work and education may particularly use telemedicine more than men. This presents an opportunity to develop culturally tailored telemedicine services specifically for Ultra-Orthodox women as part of broader efforts to expand these services within the sector. Use of telemedicine visits was found to correlate significantly and negatively with age with 41.8% users aged 30–44, compared to 16.3% aged 45–59 and 5.2% above age 60. This finding aligns with a study conducted by Jaffe et al., which examined telemedicine use in the United States during COVID-19, where adults aged 45–64 were less likely to use telemedicine than those aged 18–44 [44]. The study found that age was a significant predictor of telemedicine use compared to in-person visits. Younger individuals generally demonstrate greater willingness, experience and interest in using eHealth while older adults often have less knowledge about this technology and fear losing personal contact with their physician when starting using it. Consistent with these findings, several studies have shown that older people find using telemedicine complex [12]. Evidence from Israel similarly indicates that the use of telemedicine is higher among younger populations compared to older adults [45].

The study’s findings on income align with previous research showing that higher income individuals use eHealth more. It was found that lower income is related to limited availability and access to internet healthcare resources [38]. Furthermore, low-income patients remain less likely to use telemedicine services even when given this option [40]. Finally, in the multivariable model, education showed no significant correlation with telemedicine use after controlling for other sociodemographic variables. This finding was supported by several other studies [41].

The use of telemedicine poses significant challenges for the Ultra-Orthodox population due to limited access to technology and digital services, as well as unwillingness and fear to use the internet and participate in treatments that may conflict with their religious principles. These challenges were mentioned by the research participants as to the use of telemedicine.

Religious-ideological concerns represent the main barrier to telemedicine adoption among the study participants, with 41% of seldom/non-users reporting religious reasons and 26% reporting a lack of internet access. These findings confirm the hypothesized barriers within Ultra-Orthodox society, reflecting their complex relationship between modern technologies and traditional values. The finding that 12% of respondents were either unaware of the service or do not have the necessary technical skills required to use it highlights the potential for enhancing telemedicine adoption among the Ultra-Orthodox community through targeted education and skill programs; for instance, by collaborating with rabbinical leadership to obtain approval for ‘kosher’ digital devices specifically designated for health purposes. Furthermore, providing information and technical assistance within the workplace for Ultra-Orthodox women can increase awareness and help bridge the gap in telemedicine usage, as these women often have greater access to technology and higher digital literacy.

Many Ultra-Orthodox oppose modernity but do not reject the internet outright, using it in filtered, rabbinically approved ways to preserve their values and maintain seclusion [46]. In our study, approximately one-third of the respondents supported using restricted devices that would only access the internet for telemedicine purposes under rabbinical supervision, as a solution that can help in the adoption of telemedicine. Additionally, designated internet rooms in public spaces could facilitate telemedicine visits among the Ultra-Orthodox society and overcome the preference to avoid using the internet in their private homes.

### Strengths and Limitations

The study’s strength lies in its uniqueness as a quantitative investigation of telemedicine use within the Ultra-Orthodox population. In addition, the study is based on a representative sample of the adult Ultra-Orthodox population. As described in Section 2, the sample was drawn from a survey institute’s extensive databases, covering over 85% of the Ultra-Orthodox sector. Quota-sampling techniques were applied to ensure proper representation by gender and levels of religiosity. The representativeness of the sample is reflected in the close alignment between the study’s participants and known demographic characteristics of the wider Ultra-Orthodox population. For instance, 30.8% of the study participants held an academic degree, compared to approximately 33% in the general Ultra-Orthodox community. The marriage rate in the sample was 81%, similar to the estimated 86% of the population. In terms of family size, 47.3% of participants had between one and five children, closely matching the 48.2% found in national Ultra-Orthodox data. These similarities provide strong support for the credibility and generalizability of the study’s conclusions.

This study has some limitations. Although we asked about the use of telemedicine in medical specialties such as family medicine and pediatrics, we did not examine other medical specialties, which may impact the generalizability of the findings to specific clinical contexts. In addition, the study focused exclusively on the use of telemedicine within community settings, without addressing its use in hospitals, diagnostic institutes, or other healthcare environments.

## 5. Conclusions

While telemedicine can improve healthcare access, it may also widen public health disparities among minority populations. Therefore, it is most important to examine factors influencing the use of telemedicine among these groups in order to identify those acting as barriers for developing strategies to reduce the gaps in use. To the best of our knowledge, there are few studies on the Ultra-Orthodox society’s use of telemedicine services in Israel, and this study could contribute significant information to the field. These findings provide valuable insights for policy makers and healthcare providers in expanding the provision of telemedicine to the Ultra-Orthodox society. By identifying user characteristics and barriers, these results can assist in developing targeted education and skill-training programs designed to enhance digital literacy and telemedicine awareness within the Ultra-Orthodox community.

## Figures and Tables

**Table 1:** Distribution of sociodemographic characteristics according to use of telemedicine.

Characteristic	All n = 1460	Frequent Users n = 572	Seldom/No Users n = 888	*p* Value
	n (%)	n (%)	n (%)	
Gender				<0.001
Male	717 (49.1)	234 (40.9)	483 (54.4)	
Female	743 (50.9)	338 (59.1)	405 (45.6)	
Age categories				<0.001
18–29	628 (43)	210 (36.6)	418 (47.1)	
30–44	461 (31.6)	239 (41.8)	222 (25.0)	
45–59	258 (17.7)	93 (16.3)	165 (18.6)	
60+	113 (7.7)	30 (5.2)	83 (9.3)	
Ultra-Orthodox group				0.2
Hasidic	471 (32.3)	172 (30.1)	299 (33.7)	
Lithuanian	480 (32.9)	206 (36.0)	274 (30.9)	
Sephardic	458 (31.4)	173 (30.2)	285 (32.1)	
Other	51 (3.5)	21 (3.7)	30 (3.4)	
Marital status				<0.001
Married	1195 (81.8)	514 (89.9)	681 (76.7)	
Single	220 (15.1)	43 (7.5)	177 (19.9)	
Divorced	28 (1.9)	8 (1.4)	20 (2.3)	
Widowed	17 (1.2)	7 (1.2)	10 (1.1)	
Number of children, mean (SD)	4.1 (3.3)	4.5 (3.0)	3.9 (3.5)	<0.001
Peripherality				0.03
Central (levels 7–10)	910 (62.3)	369 (64.5)	541 (60.9)	
Peripheral (levels 1–6)	550 (37.5)	203 (35.5)	347 (39.1)	
Education				0.004
Elementary School	132 (9)	65 (11.4)	67 (7.5)	
High School	233 (16)	87 (15.2)	146 (16.4)	
Higher education (non-academic) *	598 (41)	211 (36.9)	387 (43.6)	
Academic degree	449 (30.8)	196 (34.3)	253 (28.5)	
Income (compared to the National income of Ultra-Orthodox) **				<0.001
Much below	423 (29.5)	124 (22.1)	299 (34.3)	
Below	347 (24.2)	130 (23.1)	217 (24.9)	
Similar	280 (19.5)	126 (22.4)	154 (17.7)	
Above	253 (17.7)	127 (22.6)	126 (14.5)	
Refused to answer	130 (9.1)	55 (9.8)	75 (8.6)	
House density				0.002
Low	466 (31.9)	152 (26.6)	314 (35.4)	
Moderate	755 (51.7)	320 (55.9)	435 (49.0)	
High	239 (16.4)	100 (17.5)	139 (15.7)	

* High education, non-academic—including yeshiva (religious study) for men and seminar for women. ** For income, 91 were missing data.

**Table 2:** Distribution of medical characteristics according to telemedicine use.

Characteristic	All n = 1460	Frequent Use n = 572	Seldom/Non-Users n = 888	*p* Value
	n (%)	n (%)	n (%)	
Chronic disease				0.3
Yes	129 (8.8)	53 (9.3)	76 (8.6)	
No	1331 (91.2)	519 (90.7)	812 (91.4)	
Family doctor visits in the last year				<0.001
0	374 (25.6)	112 (19.6)	262 (29.5)	
1–2	607 (41.6)	235 (41.1)	372 (41.9)	
3–5	315 (21.6)	141 (24.7)	174 (19.6)	
>5	164 (11.2)	84 (14.7)	80 (9.0)	
Expert doctor visits in the last year				<0.001
0	664 (45.5)	221 (38.6)	443 (49.9)	
1–2	553 (37.9)	223 (39.0)	330 (37.2)	
3–5	167 (11.4)	85 (14.9)	82 (9.2)	
>5	76 (5.2)	43 (7.5)	33 (3.7)	

**Table 3:** Distribution of technological characteristics by telemedicine use.

Characteristic	All n = 1460	Frequent Users n = 572	Seldom/Non-Users n = 888	*p* Value
	n (%)	n (%)	n (%)	
Mobile phone *				0.08
Simple phone	1165 (80.3)	442 (77.5)	723 (82.2)	
Kosher smartphone	151 (10.4)	65 (11.4)	86 (9.8)	
Regular Smartphone	134 (9.2)	63 (11.1)	71 (8.1)	
Mobile phone daily use *				<0.001
<2 h	857 (59.1)	301 (52.8)	556 (63.2)	
2–5	376 (25.9)	167 (29.3)	209 (23.7)	
5–8	128 (8.8)	64 (11.2)	63 (7.2)	
>8	91 (6.2)	38 (6.7)	52 (5.9)	
Availability of a computer				
Computer desk	366 (25.1)	143 (25.0)	223 (25.1)	0.9
Laptop	790 (54.1)	336 (58.7)	454 (51.1)	0.004
Internet subscription	656 (44.9)	275 (48.1)	381 (42.9)	0.05
Internet use	998 (68.4)	445 (77.8)	553 (62.3)	<0.001

* % presented without 10 subjects who reported not using a mobile phone.

**Table 4:** Multivariable logistic regression model for the association between sociodemographic, medical and technological characteristics and telemedicine use (frequent vs. seldom/no use).

	Reference Category	OR	95% CI
Age categories			
30–44	18–29	1.59	1.20–2.11
45–59	18–29	0.84	0.60–1.18
60+	18–29	0.52	0.32–0.86
Gender			
Female	Male	1.7	1.35–2.15
Ultra-Orthodox group			
Lithuanian	Hasidic	1.33	1.00–1.77
Sephardic	Hasidic	1.19	0.89–1.60
Other	Hasidic	0.97	0.50–1.85
Family status			
Married	Unmarried	2.24	1.54–3.24
Income			
Below average	Much below average	1.09	0.78–1.51
Similar to average	Much below average	1.35	0.95–1.93
Above average	Much below average	1.71	1.16–2.50
Refused to answer	Much below average	1.48	0.95–2.30
Family doctor visits			
1–2	0	1.42	1.05–1.91
3–5	0	1.9	1.35–2.67
>5	0	3.23	2.13–4.90
Mobile phone			
Internet-restricted smartphone	Regular phone	0.88	0.60–1.31
Regular smartphone	Regular phone	1.25	0.83–1.88
Internet use			
Yes	No	2.01	1.49–2.71
Internet subscription			
Yes	No	0.81	0.62–1.06

## Data Availability

The raw data supporting the conclusions of this article will be made available by the authors on request.

## Abbreviation

The following abbreviation is used in this manuscript:

UO	Ultra-Orthodox

## Appendix A

**Table A1:** Comparison of digital technological use between Ultra-Orthodox groups.

Characteristic	All * n = 1409	Hasidic n = 471	Lithuanian n = 480	Sephardic n = 458	*p* Value
	n (%)	n (%)	n (%)	n (%)	
Mobile phone					0.032
Simple phone	1138 (80.8)	399 (84.7)	383 (79.8)	356 (77.7)	
Internet-restricted smartphone	143 (10.1)	40 (8.5)	57 (11.9)	46 (10.0)	
Smartphone	118 (8.4)	31 (6.6)	36 (7.5)	51 (11.1)	
No mobile phone	10 (0.7)	1 (0.2)	4 (0.8)	5 (1.1)	
Mobile daily use					0.3
<2 h	832 (59.4)	260 (55.3)	285 (60.0)	287 (63.1)	
2–5	364 (26.0)	139 (29.6)	120 (25.3)	105 (23.1)	
5–8	118 (8.4)	43 (1.9)	41 (8.6)	34 (7.5)	
>8	86 (6.1)	28 (6.0)	29 (6.1)	29 (6.4)	
Computer desk					<0.001
Yes	354 (25.1)	97 (6.20)	152 (31.7)	105 (22.9)	
No	1055 (74.9)	374 (4.79)	328 (68.3)	353 (77.1)	
Laptop					<0.001
Yes	759 (53.9)	181 (4.38)	325 (7.67)	253 (2.55)	
No	650 (46.1)	290 (6.61)	155 (3.32)	205 (8.44)	
Internet subscription					<0.001
Yes	629 (44.6)	183 (9.38)	272 (7.56)	174 (0.38)	
No	780 (55.4)	288 (1.61)	208 (3.43)	284 (0.62)	
Internet use					0.002
No	452 (32.1)	163 (34.6)	119 (24.8)	170 (37.1)	
Yes	957 (67.9)	308 (65.4)	361 (75.2)	288 (62.9)	
Frequency of use among users **					
Seldom	70 (7.3)	25 (8.1)	17 (4.7)	28 (9.7)	
Several times a month	92 (9.6)	21 (6.8)	30 (8.3)	41 (14.2)	
1–3 times a week	162(16.9)	57 (18.4)	55 (15.2)	50 (17.4)	
Every day/almost every day	634 (66.2)	206 (66.7)	259 (71.7)	169 (58.7)	

** N = 958; * exclude “other” group.
